# Network-Based Predictors of Progression in Head and Neck Squamous Cell Carcinoma

**DOI:** 10.3389/fgene.2018.00183

**Published:** 2018-05-29

**Authors:** Nasim Sanati, Ovidiu D. Iancu, Guanming Wu, James E. Jacobs, Shannon K. McWeeney

**Affiliations:** ^1^Division of Bioinformatics and Computational Biology, Department of Medical Informatics and Clinical Epidemiology, Oregon Health and Science University, Portland, OR, United States; ^2^Department of Behavioral Neuroscience, Oregon Health and Science University, Portland, OR, United States; ^3^Division of Pediatric Hematology/Oncology, Department of Pediatrics, Oregon Health and Science University, Portland, OR, United States; ^4^OHSU Knight Cancer Institute, Portland, OR, United States

**Keywords:** HNSCC, TCGA, RNA-Seq, progression, predictors, weighted network analysis, differentially wired, co-expression

## Abstract

The heterogeneity in head and neck squamous cell carcinoma (HNSCC) has made reliable stratification extremely challenging. Behavioral risk factors such as smoking and alcohol consumption contribute to this heterogeneity. To help elucidate potential mechanisms of progression in HNSCC, we focused on elucidating patterns of gene interactions associated with tumor progression. We performed *de-novo* gene co-expression network inference utilizing 229 patient samples from The Cancer Genome Atlas (TCGA) previously annotated by Bornstein et al. (2016). Differential network analysis allowed us to contrast progressor and non-progressor cohorts. Beyond standard differential expression (DE) analysis, this approach evaluates changes in gene expression variance (differential variability DV) and changes in covariance, which we denote as differential wiring (DW). The set of affected genes was overlaid onto the co-expression network, identifying 12 modules significantly enriched in DE, DV, and/or DW genes. Additionally, we identified modules correlated with behavioral measures such as alcohol consumption and smoking status. In the module enriched for differentially wired genes, we identified network hubs including *IL10RA, DOK2, APBB1IP, UBASH3A, SASH3, CELF2, TRAF3IP3, GIMAP6, MYO1F, NCKAP1L, WAS, FERMT3, SLA, SELPLG, TNFRSF1B, WIPF1, AMICA1, PTPN22*; the network centrality and progression specificity of these genes suggest a potential role in tumor evolution mechanisms related to inflammation and microenvironment. The identification of this network-based gene signature could be further developed to guide progression stratification, highlighting how network approaches may help improve clinical research end points and ultimately aid in clinical utility.

## Introduction

Head and neck squamous cell carcinoma (HNSCC) is the most prevalent of the mucosal head and neck cancers and represents a significant health burden in the United States with approximately 40,000 new cases and almost 8,000 deaths per year. HNSCC can arise from multiple locations, including the oral cavity, oropharynx, hypopharynx, larynx, or nasopharynx (Marur and Forastiere, 2016). Furthermore, due to its non-specific presenting symptoms, patients often go undiagnosed until the cancer has progressed beyond local involvement leading to poorer treatment outcomes. Despite current treatments, many HNSCCs will become progressive which is associated with a 40–50% 5-year survival rate (Bonner et al., 2010). There are many well-known risk factors for the development of HNSCC, including smoking or chewing tobacco products, alcohol consumption, and infection with human papilloma virus (HPV). Thus, reliable stratification of patients with HNSCC given the current tumor-node-metastasis (TNM) staging system can be quite challenging with both social and biological factors at play (Cancer Staging—National Cancer Institute^1^; Patel and Shah, 2005). The ability to better predict tumor progression would be of great benefit in this patient population, allowing for the proper stratification of treatment, leading to less treatment-related morbidity in lower risk tumors as well as an increased probability of treatment success in higher risk tumors. Understanding tumor progression mechanisms is a critical step toward achieving better clinical outcomes. To this end, here we identify features predictive of tumor progression based on gene expression data.

To extract gene co-expression patterns that are predictive of tumor progression, we leverage the availability of transcriptional data coupled with clinical and behavioral measures of progression previously annotated by Bornstein et al. (2016). In their study, sample annotation with respect to progressor and non-progressor status was performed through the review and curation of The Cancer Genome Atlas (TCGA) follow-up clinical data. We hypothesize that there exist alterations in the co-expression network structure that may differentiate progressors from non-progressors. By utilizing network measures such as connectivity (pairwise gene expression correlate measures) and hubness (quantity and strength of correlation measures of a gene to all other genes), we will be able to rank the affected genes and prioritize putative predictors. These features may further aid research studies in the context of both underlying tumor progression biology and/or identifying therapeutic targets.

In order to identify mechanistically informative progressor features, we leveraged expression data to define pairwise gene relations across multiple samples as a weighted network correlation structure (Zhang and Horvath, 2005; Wu and Stein, 2012). This network model is complementary to classical approaches such as differential expression and allows for the discovery of synergies that wouldn't otherwise be evident when assessing only single gene differences. Given the complexities of tumor progression and the different dynamics observed between clinical tumor subtypes (Sparano and Paik, 2008; Hanahan and Weinberg, 2011), it is likely that the transcriptional profiles will differ between the patient group that experiences rapid tumor progression versus the group with a less aggressive disease profile. Importantly, the genetic aberrations that drive the progressor phenotype may lie in the regulatory networks between genes rather than in abnormal expression of specific gene products. Thus, network analysis is well-suited to evaluate this overall hypothesis, and in particular to detect alterations in transcriptional coordination/co-expression that are dispersed among large sets of genes and might be undetectable at the individual gene level. Similarly, relationships between transcriptional profiles and clinical and demographic variables, such as alcohol and tobacco use, could also be possibly better detected and interpreted in a network context. Finally, network measurements can also provide an alternative procedure for prioritizing and/or ranking candidates, based on their relative network importance or connectivity.

**Figure 1:**
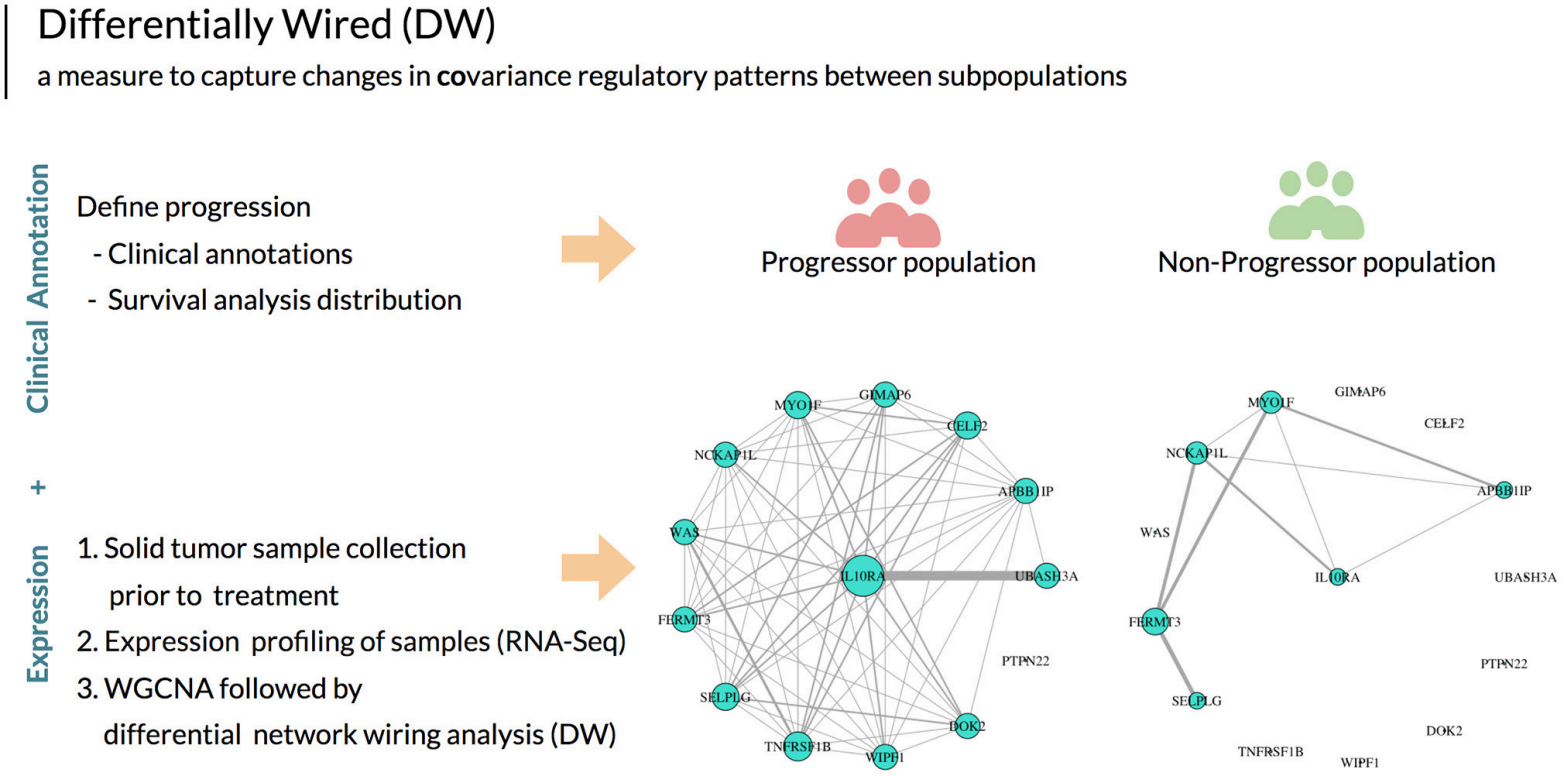
After defining disease progression based on clinical outcomes, using this annotation in aggregate with expression data and network analysis we can then utilize the unit of measure *differential wiring* (DW) to guide patient stratification. This measure detects changes in the collective transcriptional profiles of groups of genes between patient populations. These genes do not necessarily exhibit differential expression or variability. DW allows for identifying a correlation change of a gene with all other genes between two patient populations. In this example, the network edges indicate gene expression correlation measures. The size of each gene node indicates overall connectivity strength. In this example, striking differences in the co-expression networks are seen between HNSCC progressors and non-progressor for a particular gene (*IL10RA*).

When analyzing transcriptomic data between two populations, there are a variety of methods that can be utilized based on the particular hypothesis being ed. Here, we employ three measures: differential expression (DE), differential variability (DV) and differential wiring (DW) in order to identify significant aberrations that define the progressor population. In differential expression analysis (a non-network based approach), the gene expression data over multiple patient samples is used to identify genes with statistically significant differences in transcriptional status between two populations (progressor vs. non-progressor). In contrast, differential variability analysis seeks to identify genes with significant changes in variance of expression between two populations. This analysis method has been shown to identify biologically relevant genetic aberrations which can be overlooked when performing differential expression analysis alone (Ho et al., 2008). Finally, differential wiring analysis detects changes in pairwise expression correlations between genes by integrating them over all genes in the network, and selects genes and hubs enriched for these changed correlation patterns. It is important to note that such changes often affect genes that do not necessarily exhibit differential expression or variability alone (Hudson et al., 2009; Iancu et al., 2013). An example of differentially wiring (DW) between two populations is illustrated in Figure 1.

## Materials and methods

### Patient clinical demographics

Our study utilizes HNSCC data from TCGA previously annotated by Bornstein et al. (2016) (progressors and non-progressors). The data include 229 patient samples with 68 (30%) progressors and 161 (70%) non-progressors. The median last encounter days of progressor patients were considerably lower than non-progressor patients (606 vs. 4856 days). HPV status was missing in 165 (72%) patients, negative in 48 (20%), and positive in 16 (6%) (p16 or ISH). One hundred thirty-one (57%) of the tumors occurred in the oral cavity, with 59 (25%) occurring in the larynx, and 39 (17%) in the oropharynx. The mean age in the cohort was 62 years with 138 (60%) complete documented cases of self-reported tobacco pack years smoked and 97 (42%) alcohol drinks consumed per day. Remaining patients reported to be lifelong non-smokers and/or non-drinkers of alcohol or had no clinical documentation available on these two clinical features. Progressor patients had a median of 45 pack years and four alcohol drinks per day. Non-progressor patients reported slightly lower smoking and alcohol consumption estimates, with median of 40 pack years smoked and three alcoholic drinks per day.

### TCGA transcriptional data

RNA-seq data (Level 3; Illumina HiSeq, 2000) from solid tumor tissue was retrieved from TCGA. All data alignment was mapped to genome build hg19. To capture meaningful pairwise correlate measures, we utilize normalized counts per gene as defined in Li and Dewey (2011). The normalized gene counts were then transformed via a variance stabilizing log transformation *log*2(*x*+1), based on WGCNA best practices (WGCNA package^2^: Frequently Asked Questions). This transformation reduces the dependence of variance on the mean and facilitates downstream network and correlational analyses. Finally, the olfactory genes were removed from the pre-processed zero/low variance gene counts (WGCNA goodSampleGenes function). The former have been noted to introduce noise in TCGA data across cancer types due to their locations in the chromosome (Lawrence et al., 2013) and removed from multiple previous studies (Wang et al., 2014; Araya et al., 2016).

We examined the data for extreme outliers by visualizing the samples' gene expression values as boxplot distributions and computing the inter-array correlation (IAC). The IAC is defined as the average Pearson correlation of a sample to all other samples. These procedures detected no extreme outliers as all IAC had values > 0.65 (Figure S2).

### Co-expression networks construction

Following the WGCNA framework (Langfelder and Horvath, 2008), we constructed an adjacency network matrix by (1) computing the biweight mid-correlation between all gene pairs, (2) taking the absolute value of the resulting correlations for construction of an unsigned network (aggregating both up and down gene regulation), and then (3) raising this value to a power β chosen such that the network approaches a scale-free structure (exponential distribution of node connectivity; Figure S1).

Given that biological mechanisms of network components are best captured by the most connected genes, we restricted the size of the network to genes that were in the top 50% with regards to connectivity. This also reduces the overall network size and decreases the computational load while preserving scale-free topology. The resulting networks contained 10,024 genes. The adjacency matrices were further transformed to a topological overlap measure (TOM) similarity matrix. This procedure integrates information not only from the direct correlation between two gene expression patterns, but also from the correlation patterns of their network neighbors (Li and Horvath, 2007). The combination of the biweight mid-correlation coupled with the use of the topological overlap matrix transformation has been shown to outperform other measures in the discovery of biologically significant co-expression modules (Song et al., 2012).

We clustered the TOM based adjacency matrices utilizing average linkage and the WGCNA dynamicTreeCut function. Identified clusters (denoted as modules) are uniquely annotated based on their size by arbitrarily chosen colors. To preclude the emergence of highly similar modules, we further refined this procedure by examining the correlations between the module eigengenes (ME; 1st principal component). Additionally, module size was restricted to 30 genes as a lower bound to preserve any downstream statistical test assumption of normality.

Following identical procedures, separate networks were constructed utilizing the progressor and non-progressor samples. Each network's correlate weights were raised to the soft threshold powers of β = 5 and β = 6 for progressor and non-progressor conditions respectively (Figure S3).

Once we determined that modules were highly preserved across networks (see module preservation across networks), a consensus network was constructed utilizing the minimum adjacency values of the two networks, ensuring that high consensus co-expression values reflect high co-expression in both networks.

Consensus modules were detected utilizing the WGCNA blockwise Consensus Modules function, with the “max block size” parameter set to 10,024 to account for analysis of all genes in one block. This function takes a list of datasets as input, which in our case were the progressor and non-progressor samples. We utilized a dendrogram cut height of 0.995. Visualization of the resulting dendrograms and module color assignment is provided in Figures S4, S5.

### Module preservation across networks

As networks and modules were initially constructed independently in the two datasets, module overlap across networks was evaluated utilizing the WGCNA module Preservation function (Langfelder et al., 2011). This function computes module quality and preservation values. Module quality evaluates whether modules, as detected by the clustering procedure in the progressor network, significantly differ from random groups of genes in the same network. Module preservation evaluates whether modules detected in progressor network are different from random group of genes in the non-progressor network. In both cases, statistical significance is evaluated by bootsrapping (*N* = 200 permutations), which involves selecting random groups of genes of the same size as the module being evaluated. The average co-expression values of random groups of genes are then compared with the co-expression values for genes in the module and the results are returned as Z scores with associated *p*-values.

### Consensus module membership and clinical feature relationship

For each gene, we compute measures of network connectivity and gene significance. Gene connectivity is a measure of relative gene importance within a network and/or module and is quantified utilizing two measures. Intramodular connectivity is denoted as kWithin and for a gene *j* is computed with the formula $kIM_{i}=\underset{i}{\sum }\neq ja_{ij}$ where M_i_ denotes module *i* and a_ij_ denotes the adjacency between gene *j* and all other genes in module M_i_. A distinct and complementary measure of gene connectivity is quantified by the correlation between the gene expression profile and the module eigengene; this quantity is denoted as *kME* = *cor* (*x*_*i*_, *ME*). In many cases a linear relationship is expected between kME and KWithin; this relationship was observed in our data (Figure S7).

Relationships between gene expression values and clinical features are denoted as gene significance (GS) and are quantified by the magnitude of the Pearson correlation coefficient *GS*_*i*_ = |*cor* (*x*_*i*_, *F*_*clinical trait*_)|. In the present study we considered two clinical traits: tobacco use quantified as pack years, and alcohol use quantified as drinks per day. In addition to individual genes, gene modules can also be related to clinical traits; in this case the correlation is computed between the module eigengene and the clinical trait.

### Differential network analysis

Although the consensus network contains consensus modules from both conditions, there may still be statistically significant differences between the two conditions' modules, detectable at the single gene level. Ultimately, these genes may retain informative progressor differences (Fuller et al., 2007; Iancu et al., 2013). We utilize the structure and module assignment of the consensus network to provide a system level context to the changes in the transcriptional profile between the two conditions.

At the individual gene level, we quantify three distinct and complementary changes in transcription: differentially expressed (DE), differentially variable (DV), and differentially wired (DW) genes. To detect DE genes, we used the eBayes function from the “limma” R package (limma^3^; Ritchie et al., 2015). For DV genes, we compared the variance of samples utilizing the var.test function in the “stats” R package (R: F Test to Compare Two Variances^4^). Differential wiring between two genes was evaluated by examining the difference in pairwise correlation between the progressor and non-progressor groups; this approach is adapted from previous approaches of quantifying network rewiring in both genomic (Gill et al., 2010) and neural imaging studies (Hosseini et al., 2012). We utilized the r.test function in the “psych” R package. For each gene, we recorded the number of significantly changed edges (r.test *p* < 0.01). Next, we evaluated whether the number of changed edges is above what can be expected by chance, given the total number of genes and the total number of changed edges. We utilized a binomial probability with the rate $r=\frac{\#\;changed\;edges}{\#\;edges}$, i.e., the number of ***changed*** edges divided by the total number of edges in the network. Under this model the null hypothesis is that the changed edges are randomly distributed among all the nodes and the alternative hypothesis is that some nodes will be enriched in changed edges. In the binomial distribution each gene has N trials, where N is the number of genes and also the number of edges for each gene. The probability of a gene having at least *n*_*i*_ changed edges is *p*(*n* > *n*_*i*_) = *B* (*N, r*). The set of DW genes was then taken to be the genes enriched in changed edges at *p* < 0.01.

We denote as “affected genes” the set of genes that are DE, DV, or DW. Next, we inquired whether individual modules contain more of the affected genes than what can be expected by chance given the size of the affected gene set, size of the network and the size of the module (Fisher's exact test). Bonferroni corrections were done based on the number of modules.

### Pathway enrichment analysis

Consensus modules' pathway enrichment analysis was assessed utilizing ReactomeFIViz, the Reactome Cytoscape app (ReactomeFIViz—ReactomeWiki^5^; Wu et al., 2014). ReactomeFIViz's basic data entities are known proteins and their functional interactions. The overall data network model is designed to capture known functional protein-protein interactions (PPI). This analysis was applied to the full gene set of all progressor consensus modules including those enriched in affected genes. Detailed pathway enrichment analysis tables (pathway, Binomial *p*-value, FDR, gene list) along with co-expression consensus modules gene lists (utilizable as input to ReactomeFIViz) can be found in Supplementary Data-2, 3, 4.

### Software

The R code for this project is open-source and can be accessed via https://github.com/teslajoy.

## Results

### Progressor network construction and preservation

**Figure 2:**
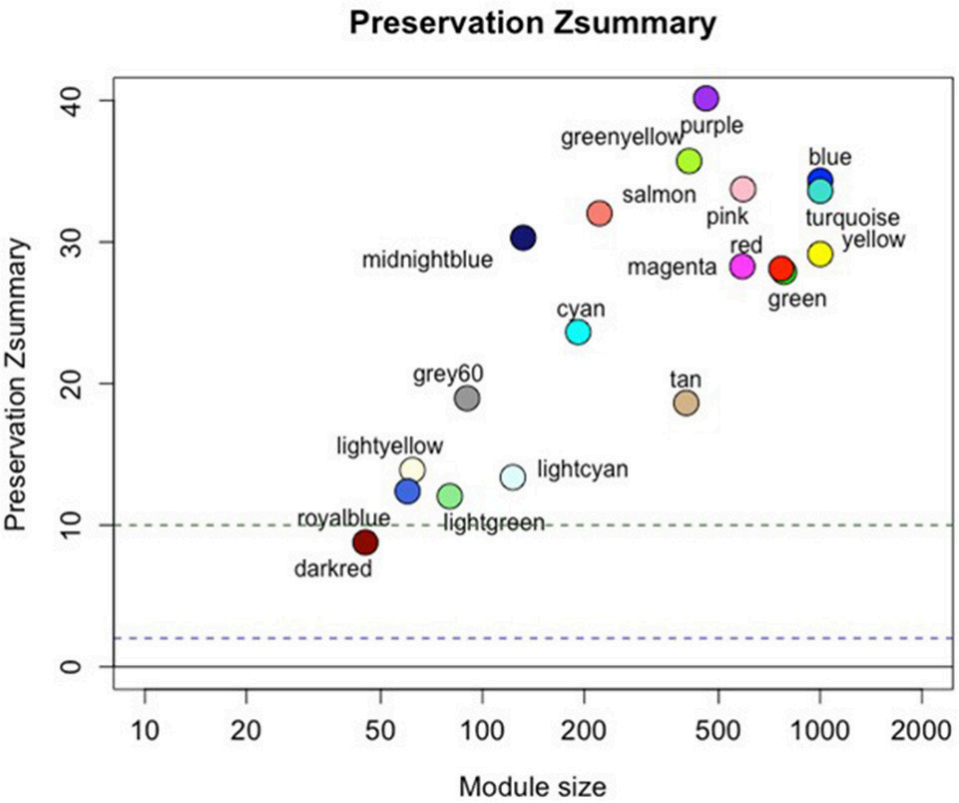
Progressor network modules are preserved in the non-progressor network. Figure shows *preservation Z*_*summary*_ statistic (y-axis) as a function of module size. The dashed blue (low) and green (high) lines are thresholds highlighting 2 < Z < 10 region corresponding to moderate/high preservation. Detailed statistics of all modules are listed in the Supplementary Data-1.

We constructed a co-expression network based on the progressor samples, identifying 21 modules ranging in size from 45 to 1,127 genes. Modules were evaluated for both quality (Figure S4) and preservation in the non-progressor network (Figure 2—see also Supplementary Data-1). All progressor modules displayed high quality measures and also showed high preservation in the non-progressor weighted network (Figure 2; Supplementary Data-1).

### Consensus network construction

The preservation of the progressor modules in the non-progressor network indicates that overall network and module structure is preserved and no progressor-specific modules have emerged. In light of this finding, we combined the two conditions' datasets and constructed clustering of consensus modules. Network adjacency in the consensus network was based on the minimum adjacency across the two conditions.

**Figure 3:**
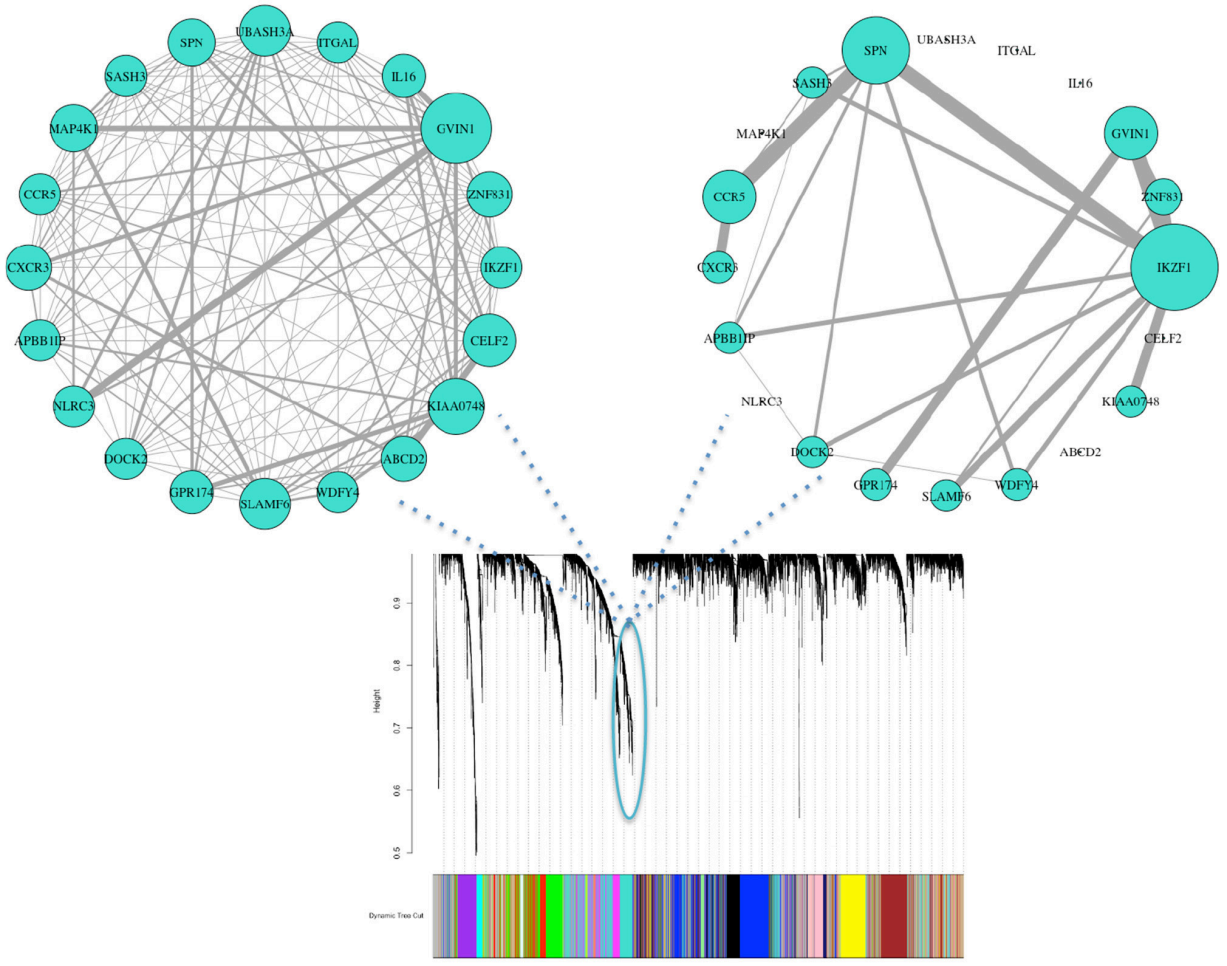
Consensus clustering identifies organized gene expression patterns of both progressor (**left**) and non-progressor (**right**) conditions. These clusters are subnetworks of tightly connected nodes (genes) that we refer to as modules. Here we demonstrate one of the modules color-coded in turquoise, with clear wiring differences/variability (gene effects) between the two conditions. Nodes are the top 20 genes with high kME measure in progressor condition (45% have high kME in non-progressor). Wiring width demonstrates correlation magnitude strength. The networks' wiring weights are correlations greater than 0.6. Size of each genes node indicates overall connectivity strength.

Biological consensus modules between the two conditions are made of similar gene structure but their interactions and expressions are variable (Figure 3). Also, since each consensus module retains different samples, progressors and non-progressors separately, their module properties are not the same (e.g., module eigengene).

We identified 18 consensus modules ranging from 71 to 1,389 genes (Figure 3, Table S1). There were 882 unclustered genes that were assigned to the “gray” pseudo module. All genes showed high module membership with kME and KIM Pearson correlations > 0.9 (*p* < 0.01; Figure S6).

### Relating smoking and alcohol exposure to consensus modules

**Figure 4:**
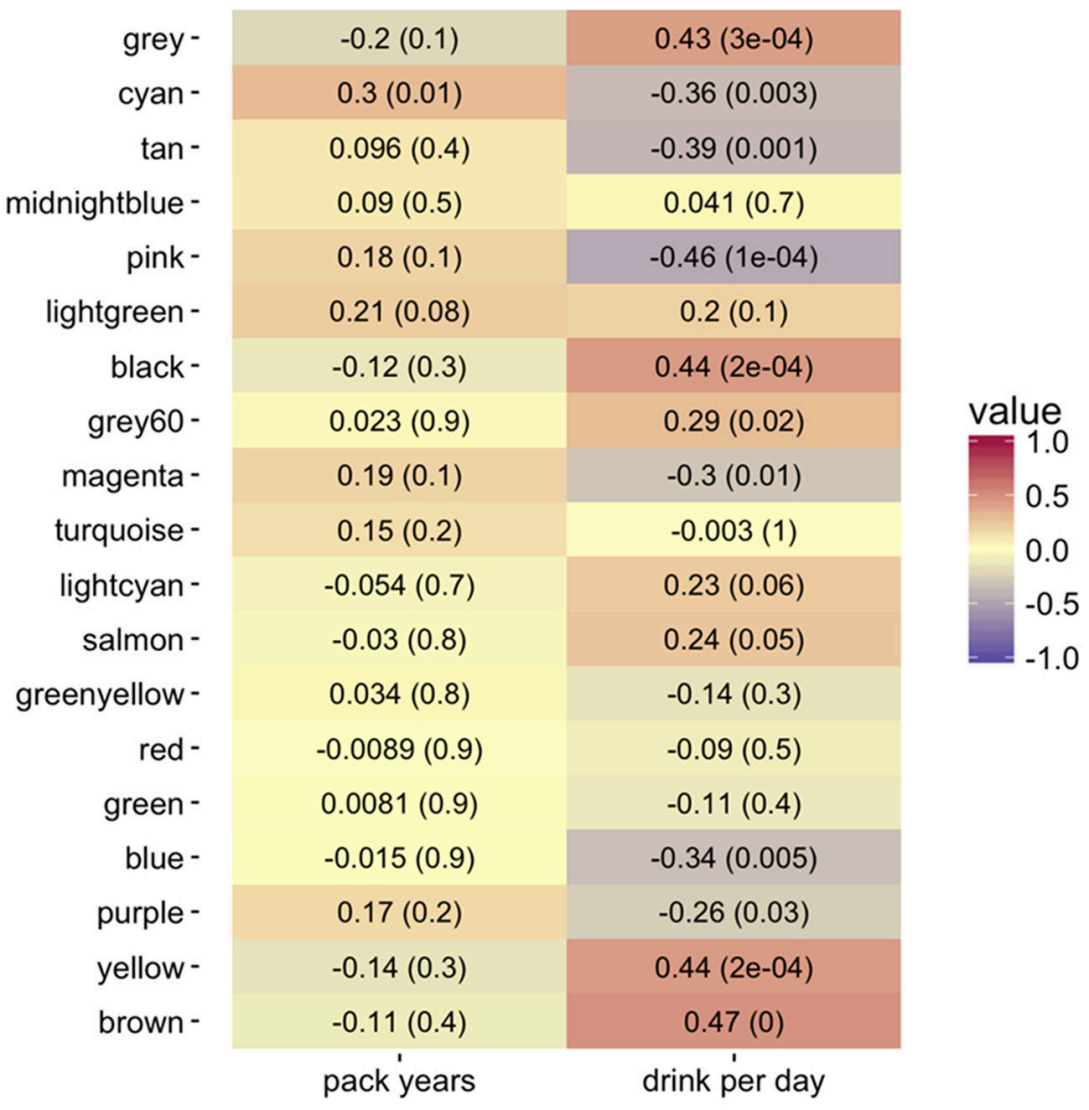
Heatmap of Pearson correlations (−1:1 shown by color legend) for alcohol (drinks per day) and tobacco use (pack years) with the co-expression consensus module progressor eigengenes. The corresponding *p*-values are in parentheses. The brown, yellow, black, pink, tan, and cyan modules show the highest positive correlation with drinks per day. The cyan module shows the highest positive correlation with tobacco pack years smoked.

We evaluated the relationship between gene expression and tobacco/alcohol use both at the level of individual genes and at the level of gene modules. At the individual genes, module membership (as measured by kME) showed strong correlation with gene significance for our clinical risk factors (see Figures S7–S9). At the module level, we correlated the module eigengenes to the same “pack years” and “drinks per day” risk factors. We utilized the consensus module assignment, but eigengenes were constructed separately for the progressor and non-progressor networks. While the non-progressor module eigengenes showed only weak correlations with each risk factor, in the progressor network, seven module eigengenes had a stronger relationship with alcohol consumption; in contrast only the cyan module had a strong correlation with tobacco use (Figure 4).

### Cancer progression and differential network analysis

Leveraging network properties at the individual gene level, we quantified differences in expression, variability, and correlation (DE, DV, and DW respectively); each measures' gene list is available via the Supplementary Data-3.

Not all modules had an equal proportion of affected genes. We identified 7,055 genes that were differentially expressed, 3,682 genes that were differentially variable, and 34 genes that were differentially wired. Overall, 12 modules were enriched in identified affected genes, with 11 having more DE or DV genes and only the turquoise module having above the expected DW genes (Table 1). All identified enriched modules' expression profiles were evenly distributed and showed no outstanding noise/batch effect (Figure S10).

**Table 1:** Summary of identified consensus modules enriched in affected genes via differential network analysis between progressor and non-progressor conditions.

**DW**	**DV**	**DE**
Turquoise (14)	Black (242)	Black (513)
	Purple (212)	Blue (1298)
	Pink (236)	Cyan (148)
	Light cyan (71)	Tan (215)
	Salmon (95)	Gray60 (71)
		Yellow (658)
		Light green (69)

*The number of affected genes found in each module is noted in parentheses*.

**Figure 5:**
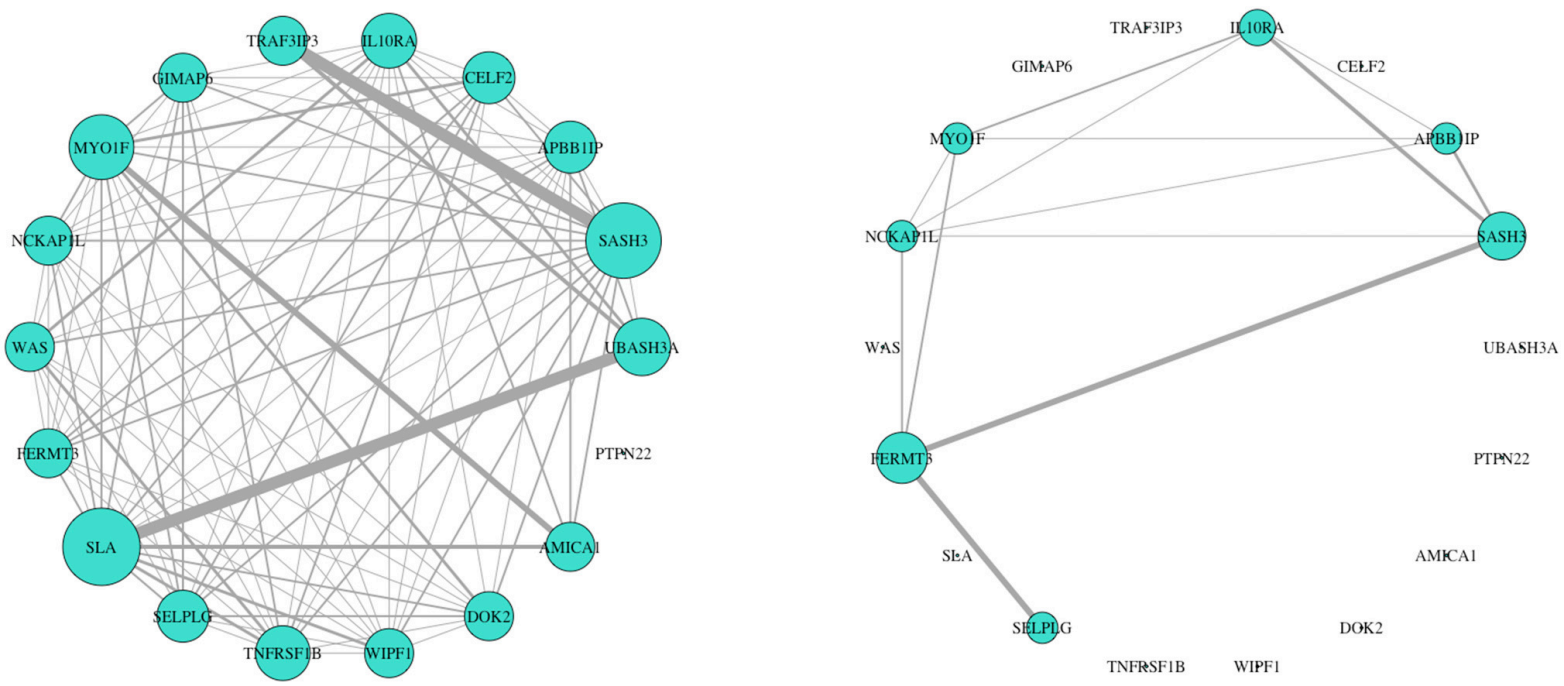
Network structure of 18 DW hub genes showed striking expression connectivity measure differences between HNSCC progressor (left) and non-progressor (right) conditions. These genes were identified as differentially wired hub nodes in the turquoise module [kME > 0.8 and enriched in changed edges (*p* < 0.01; Binomial)]. Visually, wiring width demonstrates correlation magnitude strength. Weights are correlations > 0.6 to capture mid/low strength connectivity in the non-progressor condition. The size of each gene's node indicates overall connectivity strength.

We further analyzed connectivity and hubness (potential driver events) of DW genes in the turquoise module. From the 34 DW genes, we identified 18 genes (shown in Table 2) that were both hubs (kME > 0.8) and enriched in changed edges (*p* < 0.01; Binomial). The visual network structure and wiring differences of these 18 genes between HNSCC progressor and non-progressor conditions can be seen in Figure 5. This result confirms our hypothesis on the existence of alterations in the co-expression network structure that differentiate progressor from non-progressor populations.

**Table 2:** Summary measure of 18 genes identified as differentially wired hub genes in the turquoise module [kME > 0.8 and enriched in changed edges (*p* < 0.01; Binomial)].

**DW Genes**	**kME**	**Changed edge enrichment *p*-value**
*UBASH3A*	0.9378	2.4596e-04
*SASH3*	0.9325	1.8283e-06
*APBB1IP*	0.9278	2.4596e-04
*CELF2*	0.9205	1.7880e-40
*IL10RA*	0.9054	2.4596e-04
*TRAF3IP3*	0.9005	1.8283e-06
*GIMAP6*	0.8985	1.5076e-18
*MYO1F*	0.8969	2.4596e-04
*NCKAP1L*	0.8965	2.4596e-04
*WAS*	0.8943	2.4596e-04
*FERMT3*	0.8919	2.4596e-04
*SLA*	0.8846	2.4596e-04
*SELPLG*	0.8813	2.4596e-04
*TNFRSF1B*	0.8745	1.0199e-08
*WIPF1*	0.8677	1.8283e-06
*DOK2*	0.8419	2.3464e-43
*AMICA1*	0.8368	8.6198e-35
*PTPN22*	0.8226	2.4596e-04

*The genes are sorted by the magnitude of kME*.

### Pathway enrichment analysis of enriched modules

We performed a pathway enrichment analysis of the modules enriched in DE/DV/DW affected genes. To capture known pathways, we included all genes of enriched modules.

Pathway enrichment analysis of the progressor module enriched in DW genes showed involvement in inflammation, T cells, B cells, natural killer cells, adhesion, and the Jak-Stat pathway. This result adds to the evidence in the Bornstein et al. study (2016) and reemphasizes the potential importance of cancer associated inflammation pathways and tumor microenvironment evolutionary mechanisms in tumors with higher progression rates. We also found the pathway association between the identified 18 differentially wired hub genes has not been discovered and remains unknown.

Modules enriched in DE or DV genes showed involvement in multiple hallmarks of cancer and tumor mechanism dynamics such as cell cycle check points, abnormal mitosis, spindle bipolarity, receptor synapse deregulation and important cellular pathways such as c-myc, MAPK, Jak-Stat, and P53. There were also noted associations between the enriched pathways and diseases such as Alzheimer's and Parkinson's. This characterized information could potentially further our research on tumor pathway completion studies.

We also assessed the overlap of enriched modules in pathways with FDR < 0.05. This was to investigate the co-dependency or overlap of biological mechanisms despite the measure of affected genes (Table 3). We found 149 pathways that overlapped between modules and 16 pathways that involved DV or DW enriched modules. The affected genes were identified to be unique to each associated pathway, but overlapping between pathways. Notably, pathways associated with DW measure show high involvement of inflammation and tumor microenvironment evolution mechanisms.

**Table 3:** Summary of pathway enrichment analysis showing pathways (FDR < 0.05) that overlapped between modules and are enriched in genes showing DV/DW.

**Pathway**	**Modules**	**Measure**	**FDR**	**Genes**
Chemokine signaling pathway(K)	Turquoise, salmon	DW, DV	0, 0.002	*GNG2, PIK3CG, NCF1, GNGT2, LYN, WAS, CCL25, CCL22, CCL21, ARRB1, CX3CL1, CCL5, DOCK2, VAV1, CXCL13, XCL2, RAF1, JAK2, JAK3, ADCY6, CCR8, CCR7, CCR6, CCR5, CCR4, CX3CR1, CCR2, XCR1, CXCL9, STAT3, ITK, CXCR3, PLCB3, CXCR4, CXCR6, PLCB2, PRKCB, RASGRP2, PIK3R5, PIK3R1, BCAR1, CXCR5, PIK3R3, GNG7, CCL19*
Platelet activation(K)	Turquoise, pink	DW, DV	5e-04, 0.038	*BTK, ACTG1, PIK3CG, PPP1CA, LYN, APBB1IP, P2RY12, MYL12B, P2RX1, LCP2, ADCY6, PLA2G4C, ITPR1, GP5, PLCB3, PLCB2, RASGRP1, RASGRP2, PIK3R5, PIK3R1, MAPK3, GNAI3, GNAI1, PLA2G4F, PLA2G4E, PLA2G4D*
B cell receptor signaling pathway(K)	Turquoise, salmon	DW, DV	6e-04, 0	*BTK, PIK3CG, NFKBIE, RAC3, LYN, CARD11, VAV1, INPP5D, NFATC2, RAF1, PTPN6, PRKCB, PIK3R5, PIK3R1, CD22, PIK3R3, CR2, CD19, CD79B, CD79A*
BCR signaling pathway(N)	Turquoise, salmon	DW, DV	8.00E-04, 8.00E-04	*BTK, LYN, CARD11, RASA1, INPP5D, RAF1, MAP4K1, PTPN6, MAP3K1, POU2F2, PIK3R1, PTPRC, SH3BP5, CD22, CD19, CD79B, CD79A*
Fc gamma R-mediated phagocytosis(K)	Turquoise, pink	DW, DV	0.0018, 0.038	*PIK3CG, NCF1, SPHK2, LYN, WAS, DOCK2, VAV1, LAT, PIP5K1B, INPP5D, RAF1, PRKCB, PIK3R5, PIK3R1, PTPRC, PLD2, MAPK3, PLA2G4F, PLA2G4E, PLA2G4D*
GPCR downstream signaling(R)	turquoise, light cyan	DW, DV	0.011, 0.0438	*GNG2, PIK3CG, SSTR3, GAST, RGS1, NMUR1, ARHGEF4, ARHGEF6, GPR65, CCL25, CCL21, P2RY12, P2RY13, P2RY10, P2RY14, PDE3B, PDE4D, GPR55, FGD2, DRD1, CYSLTR1, ADORA2A, CCL5, ADRA2A, FGD3, VAV1, CXCL13, XCL2, CSF2RB, RHOB, CSF2RA, GPR18, JAK2, JAK3, ADCY6, S1PR4, GPR132, CCR8, CCR7, CCR6, CCR5, CCR4, CCR2, ABR, CCKAR, OXER1, XCR1, CXCL9, NMB, ITPR1, VIPR2, PTHLH, IL3RA, INSL3, CXCR3, ARHGAP4, PLCB3, CXCR4, CXCR6, PLCB2, RAMP3, GHRL, RASGRP1, RASGRP2, IL2RG, PIK3R5, PIK3R1, IL2RB, GNA14, TACR1, HTR2B, MCF2L*
Oxytocin signaling pathway(K)	Turquoise, purple, pink	DW, DV	0.0159, 0, 0.0024	*ACTG1, PIK3CG, PPP1CA, KCNJ2, CACNA2D4, CACNA1F, NFATC2, RAF1, ADCY6, CAMK4, PLA2G4C, ITPR1, PLCB3, PLCB2, PRKCB, PPP1R12B, PIK3R5, PIK3R1, MEF2C, ADCY2, MYLK3, MYLK2, RYR1, CACNB1, CACNA1S, PRKAG3, CACNG6, CACNG1, CAMK2B, PRKAA2, CAMK2A, PRKAB1, MAPK3, GNAI3, GNAI1, ELK1, CDKN1A, PLA2G4F, PLA2G4E, PLA2G4D, CALM1*
Ras signaling pathway(K)	Turquoise, pink	DW, DV	0.0183, 0.0206	*GNG2, PIK3CG, RAC3, ZAP70, GNGT2, RASA1, RASSF5, RGL1, MET, LAT, RRAS2, RAF1, EFNA3, PLA2G4C, FOXO4, STK4, RASAL3, FASLG, PRKCB, RASGRP1, RASGRP2, PIK3R5, PIK3R1, GAB1, PLD2, MAPK3, PLA2G2F, ELK1, PLA2G4F, PLA2G4E, PLA2G4D, CALM1*
Gastrin-CREB signaling pathway via PKC and MAPK(R)	Turquoise, pink, light cyan	DW, DV	0.026, 0.0129, 0.0127	*GNG2, GAST, NMUR1, GPR65, P2RY10, CYSLTR1, XCL2, RAF1, JAK2, GPR132, CCKAR, XCR1, NMB, ITPR1, PLCB3, PLCB2, GHRL, RASGRP1, RASGRP2, PIK3R1, GNA15, LTB4R, LPAR5, MAPK3, BDKRB1, BDKRB2, LTB4R2, GNRHR, ANXA1, GNA14, TACR1, HTR2B*
Neurotransmitter Receptor Binding And Downstream Transmission In The Post-synaptic Cell(R)	Turquoise, purple	DW, DV	0.0295, 0.0049	*GNG2, KCNJ2, GNGT2, GRIK5, RAF1, ADCY6, CHRNA7, CHRNA6, CAMK4, KCNJ10, AKAP5, PLCB3, PLCB2, PRKCB, MDM2, ADCY2, CHRNA1, ACTN2, CHRND, CHRNG, CAMK2B, CAMK2A*
VEGF signaling pathway(K)	Turquoise, pink	DW, DV	0.0334, 0.0451	*PIK3CG, RAC3, SPHK2, NFATC2, RAF1, PLA2G4C, PRKCB, PIK3R5, PIK3R1, MAPK3, PLA2G4F, PLA2G4E, PLA2G4D*
Gastric acid secretion(K)	Turquoise, purple	DW, DV	0.0354, 2e-04	*GAST, KCNJ2, ADCY6, KCNJ10, ATP1A3, ATP1A4, ITPR1, PLCB3, PLCB2, PRKCB, ADCY2, ATP1B4, MYLK3, MYLK2, CAMK2B, CAMK2A, ATP1A2*
Central carbon metabolism in cancer(K)	turquoise, pink	DW, DV	0.0494, 0.0176	*PIK3CG, TP53, SLC7A5, SLC2A1, MET, FLT3, RAF1, PIK3R5, PIK3R1, PGAM1, PGAM4, HIF1A, MAPK3, HK2*
Calcium signaling pathway(K)	purple, pink, light cyan	DV	0, 0.0451, 5e-04	*SLC8A3, ADCY2, TNNC2, TNNC1, ATP2B2, MYLK3, MYLK2, RYR1, CACNA1S, ATP2A1, PHKG1, CAMK2B, CAMK2A, PLN, GNA15, ITPKA, BDKRB1, BDKRB2, PPIF, LTB4R2, CALM1, GNA14, TACR1, HTR2B, CACNA1D*
Glutamatergic synapse(K)	pink, salmon	DV	0.0024, 0.0437	*PLD2, MAPK3, GNAI3, GNAI1, HOMER1, HOMER2, PLA2G4F, PLA2G4E, PLA2G4D, HOMER3, GNG7, GRM7*
Serotonergic synapse(K)	pink, light cyan	DV	0.0122, 0.0438	*MAPK3, GNAI3, GNAI1, PLA2G4F, PLA2G4E, PLA2G4D, ALOX12B, HTR2B, CACNA1D*

*In order to highlight findings from the network analysis, DE modules have been excluded. Genes of the associated pathway are unique within each pathway, but overlap between pathways (Supplementary Data-5)*.

## Discussion

Previously annotated and curated TCGA HNSCC data (Bornstein et al., 2016) provides the opportunity to study samples from both progressors and non-progressors from a network perspective. Weighted networks analysis can provide a holistic view on disease dynamics, but also enables us to reduce the complexity into organized and measurable relations. We were able to reduce the expression data from over 10,000 genes to 18 mechanistic modules. Within these modules we further focused on gene hubs with high correlation with alcohol/tobacco use, or with changing network profiles. Finally, through pathway enrichment analysis, we provide a context for the activity of these genes and relate them to biologically relevant pathways through the human protein Interactome.

Among the modules enriched in DE/DV/DW genes we identified aberrations in molecular pathways important for cell cycle check points, mitosis and spindle bipolarity, macrophage activity, immune response and T cells, interferon gamma signaling pathway, as well as c-myc, MAPK, Jak-Stat, and P53 pathways (see Supplementary Materials for a complete list of affected pathways). More specifically, pathway enrichment analysis of the module enriched in DW genes highlighted involvement of inflammation and tumor microenvironment evolution mechanisms. These results are an important extension to previous finding of Bornstein et al. (2016). We identified *IL10RA, DOK2, APBB1IP, UBASH3A, SASH3, CELF2, TRAF3IP3, GIMAP6, MYO1F, NCKAP1L, WAS, FERMT3, SLA, SELPLG, TNFRSF1B, WIPF1, AMICA1, PTPN22* as differentially wired hub genes (putative network driver genes). This putative signature represents an early step toward developing stratification techniques based on tumor genomics that could potentially identify patients with a high likelihood of tumor progression early on. Interestingly, it has been shown that gene expression networks show characteristic changes in correlation during transition from normal to a disease state (Censi et al., 2011) as well as during normal cellular differentiation (Mojtahedi et al., 2016). Given these measurable changes in network properties and the ability of differential wiring analysis to provide a global descriptor for the network, it is feasible that—with more samples—a gene expression network threshold could be defined for the progressor phenotype. Utilized as a surveillance tool, this could provide clinicians with an early warning for patients at risk of developing progressive disease. Furthermore, if the progressor-specific molecular pathways that we have identified are validated in cell and animal models, our findings could aid in the development of targeted therapy for HNSCC.

We also assessed the overlap of significantly enriched modules in pathways (Table 3). We found 149 pathways that overlapped between modules and 16 that overlapped modules specifically enriched in DV/DW genes. The affected genes were identified to be unique to each associated pathway, but overlapping between pathways. Given the clinical phenotype of interest, progression, pathways associated with DW measure show high involvement of inflammation and tumor microenvironment evolution mechanisms. The identification of Ras signaling as a pathway that overlaps with multiple modules enriched in DV/DW genes serves as a validation of this type of network analysis in tumor genomics. This well-known cellular signaling pathway is involved in critical functions such as cell growth, migration, adhesion, cell survival, and cell differentiation (Rajalingam et al., 2007; Fernández-Medarde and Santos, 2011). Aberrations in the Ras pathway are some of the most frequent findings in human cancers and have been previously described in some HNSCC samples (Hoa et al., 2002; Rothenberg and Ellisen, 2012). Interestingly, in colorectal cancer, the EGFR antibody, cetuximab, showed benefit in clinical trials, but the benefits did not hold for patients with Ras mutations. Thus, the development of targeted agents for the Ras pathway is an active area of research (Bahrami et al., 2017; Simanshu et al., 2017). Also of interest is the identification of the VEGF signaling pathway. This family of growth factors is involved in angiogenesis and its members have been shown to be involved in a variety of human malignancies (Olsson et al., 2006; Stacker and Achen, 2013). Furthermore, previous studies have linked VEGF signaling to HNSCC (Tong et al., 2008; Lucas et al., 2010). As therapeutic options are currently limited in HNSCC, our finding adds strength to the evidence that VEGF signaling may be a potentially targetable pathway in HNSCC.

The current focus of therapeutic advancement in cancer calls for a precision/personalized approach based on detectable molecular abnormalities of a patient's tumor that can then be exploited in a targeted manner. However, many questions still remain open as to what the best methods of molecular analysis are, who is likely to benefit, and why. Our results suggest that a stratification procedure could benefit from inclusion of transcriptional hub genes related to disease progression. Additionally, DW measure assessment over temporal data could potentially be informative regarding targeted therapy failure or drug outcome predictions between subpopulations which has not been clinically evaluated (e.g., currently we have only assessed EGFR and cetuximab). If this measure is captured longitudinally, it has the potential to reveal novel discoveries on disease state and tumor evolution mechanisms.

Beyond progression status, we also evaluated gene expression correlations with alcohol and tobacco use. Although data annotation of alcohol consumption per day was less complete than pack years, we found stronger associations of alcohol habits with co-expression consensus modules (Figure 4; Figure S9). We hypothesize that this is potentially due to patients' stronger recall on the quantity they drink per day vs. packs of cigarettes they smoke through a year. This finding underlies the importance of advancing the quality of measured clinical data for improving research results.

Overall we have shown that the use of *de-novo* weighted network inference in the context of biological pathways provides new insights for both mechanistic and prognostically relevant information in HNSCC. Follow-up studies can incorporate other clinical phenotypes such as measurements derived from tumor imaging and could ultimately lead to a greater understanding of these tumors.

## Author contributions

NS conceived of the study and developed or extended code, performed analysis and wrote the majority of the manuscript. GW, OI, and SM provided input into the design and analyses. JJ provided clinical perspective. GW, OI, SM, and JJ provided guidance on presentation of data and contributed to editing of the manuscript.

### Conflict of interest statement

The authors declare that the research was conducted in the absence of any commercial or financial relationships that could be construed as a potential conflict of interest.

## Footnotes

^1^Cancer Staging—National Cancer Institute. Available online at: https://www.cancer.gov/about-cancer/diagnosis-staging/staging (Accessed August 4, 2017).

^2^WGCNA package: Frequently Asked Questions Available online at: https://labs.genetics.ucla.edu/horvath/CoexpressionNetwork/Rpackages/WGCNA/faq.html (Accessed December 20, 2016).

^3^limma Bioconductor. Available online at: http://bioconductor.org/packages/limma/ (Accessed September 2, 2017).

^4^R: F Test to Compare Two Variances Available online at: https://stat.ethz.ch/R-manual/R-devel/library/stats/html/var.test.html (Accessed September 2, 2017).

^5^ReactomeFIViz—ReactomeWiki Available online at: http://wiki.reactome.org/index.php/ReactomeFIViz (Accessed September 2, 2017).
