# Supplementary material for: Network-Based Predictors of Progression in Head and Neck Squamous Cell Carcinoma
Source: Front Genet. 2018 May 29;9:183. doi: 10.3389/fgene.2018.00183 (PMC5992410; doi:10.3389/fgene.2018.00183)
Supplement: Supplementary file 2 [file Presentation_1.pdf]

Supplementary –Network EDA Results

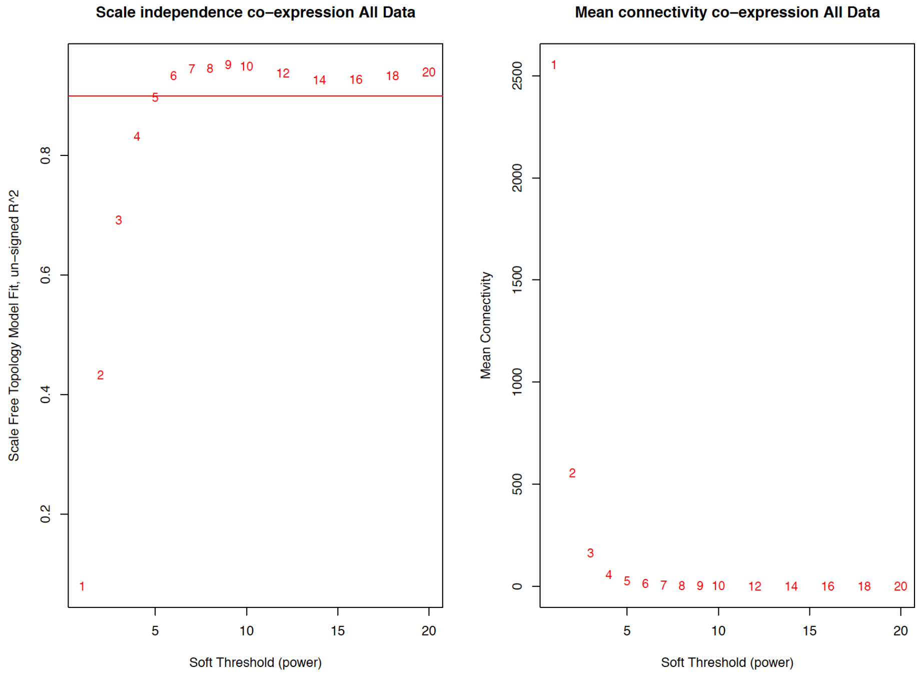

Figure S1: All data co-expression beta estimates. Left plot is the linear model-fitting index R squared over possible soft thresholds to choose from. Red line is at R squared threshold of 0.85. Right plot shows mean connectivity over possible soft thresholds (beta) to aid in preserving information and account for node connectivity measures. Beta estimate of 5 was evaluated by pickSoftThreshold WGCNA function.

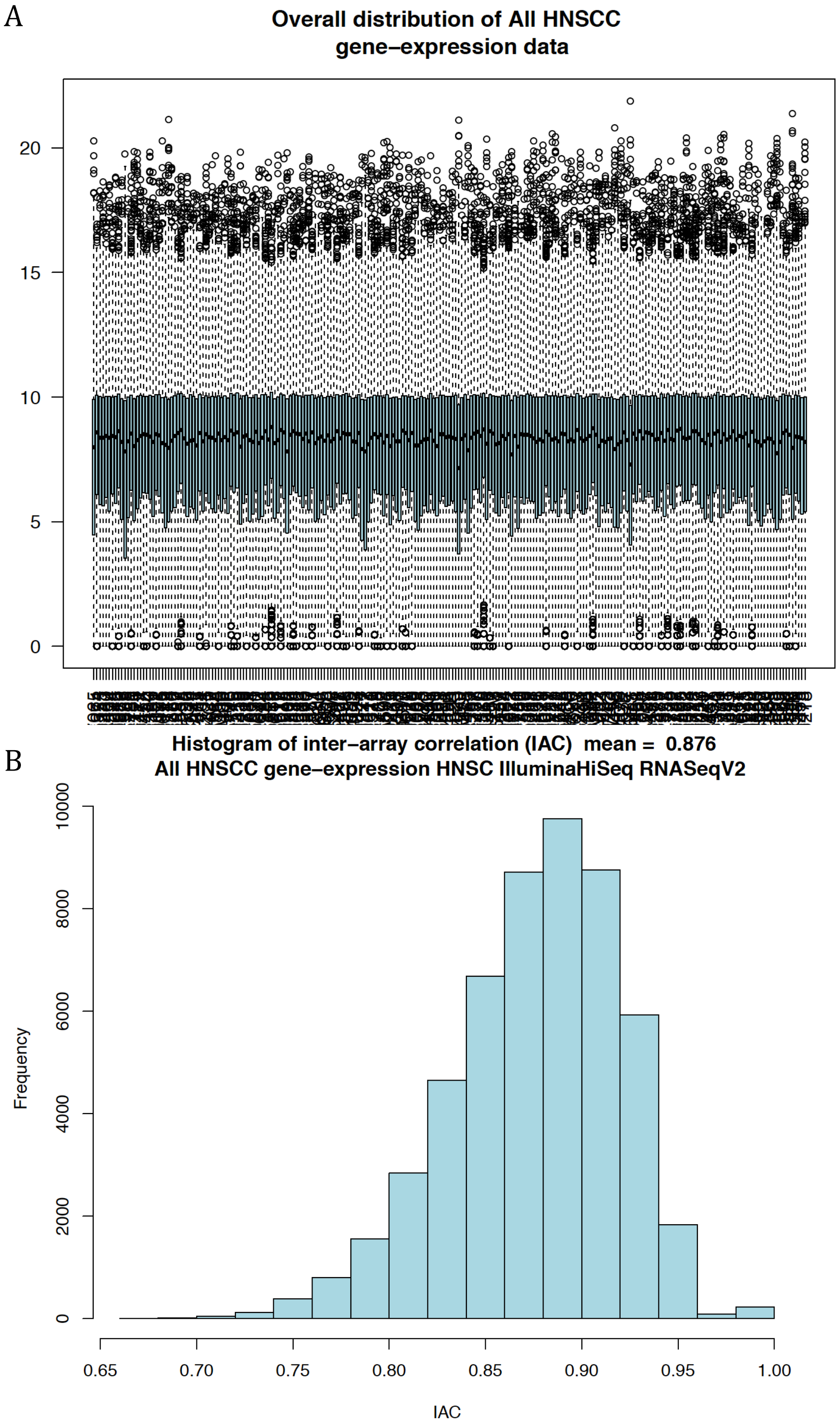

Figure S2: A) Boxplot distribution of all samples RNA_SeqV2 gene expression normalized counts that are log transformed and shifted log2 (x + 1). B) Histogram distribution of all samples Inter Array Correlation demonstrating each gene profile (gene in all samples vector) correlation value frequencies. RNA_SeqV2 gene expression normalized counts that are log transformed and shifted log2(x + 1). The goal is to reduce the long tail on left that is indicative of low correlations and suggestive of possible outliers.

Supplementary – Network Construction and Results

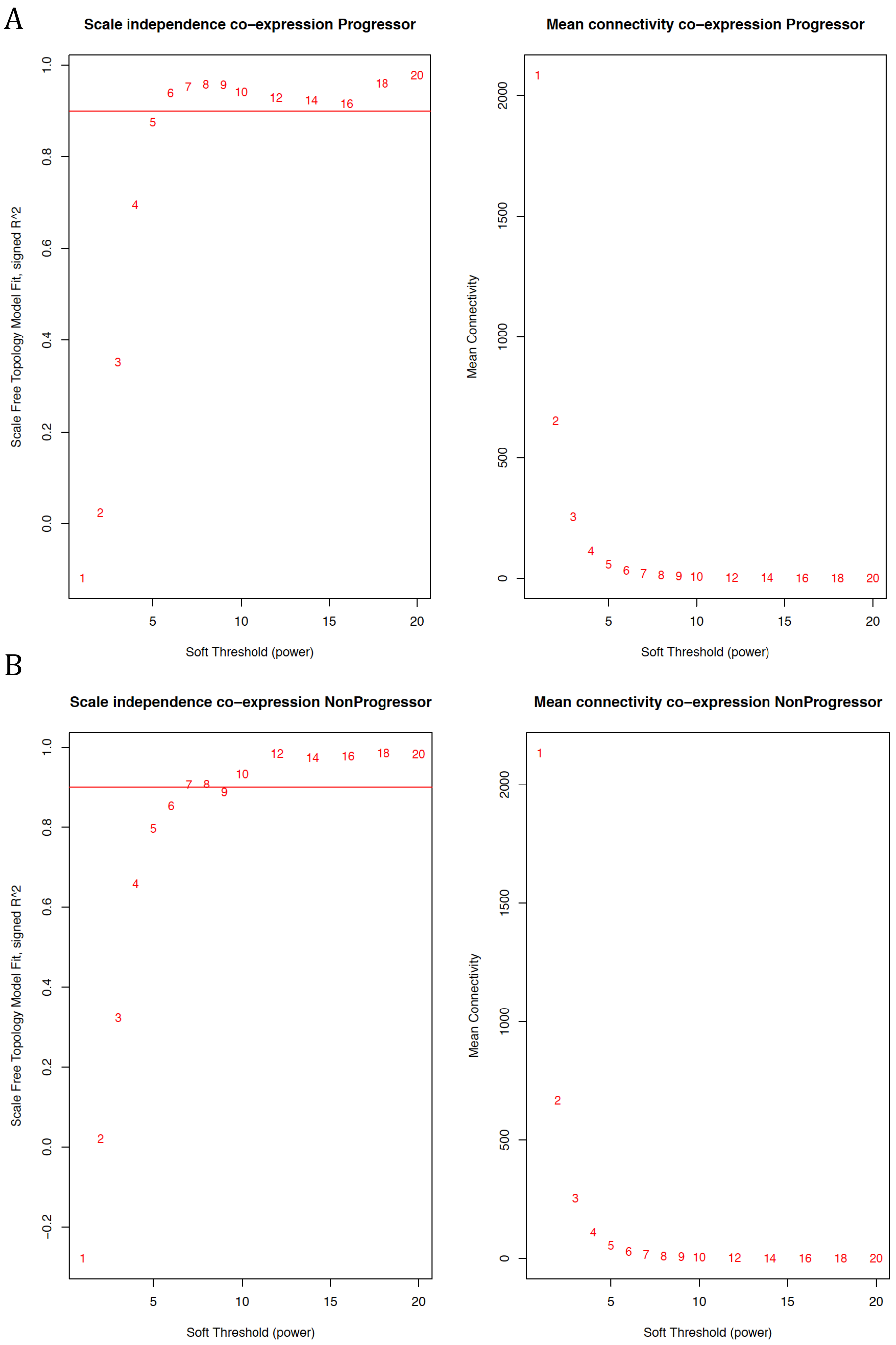

Figure S3. Progressors co-expression (A) Nonprogressors co-expression (B) Beta estimate of A) 5 and B) 6 were evaluated by pickSoftThreshold WGCNA function.

A

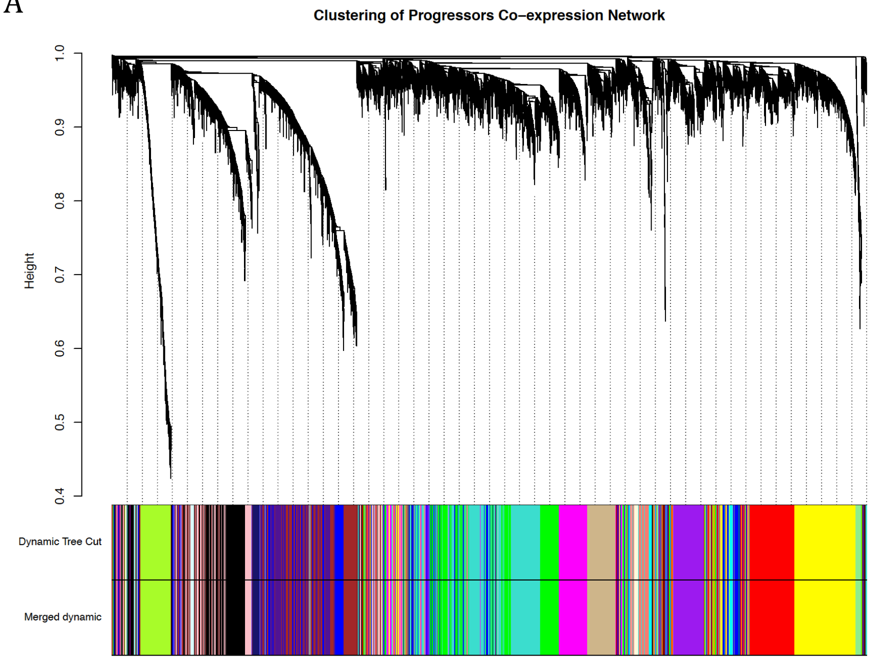

B

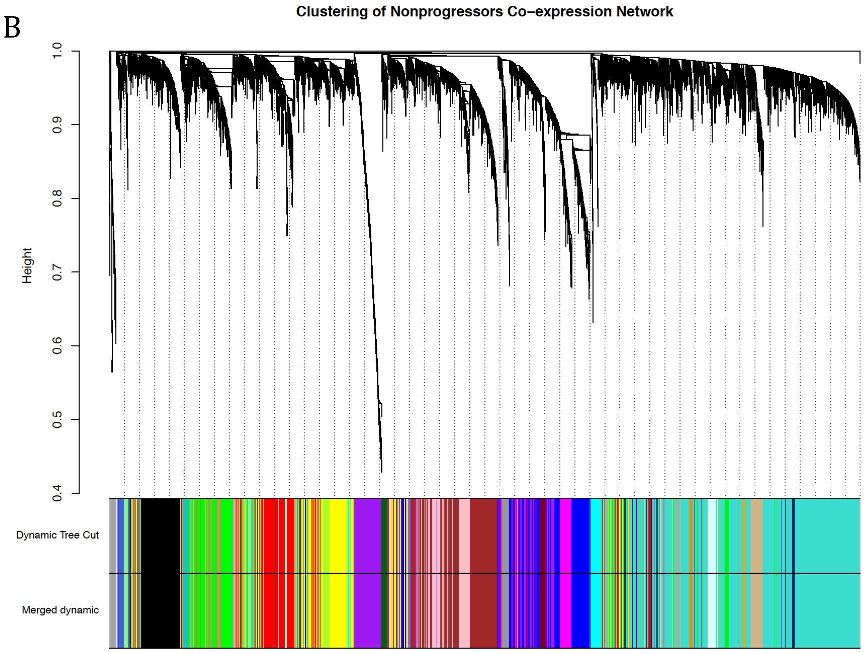

Figure S4: *Progressor (A) and NonProgressor (B) RNA_SeqV2 normalized counts and log2 (x+1) transformed gene expression* dynamic clustering and module definition denoted by first color-band.
Second color band studies the need of merging similar modules based on similarity of original modules eigengenes (1st principal component). Here merging was clearly unnecessary.

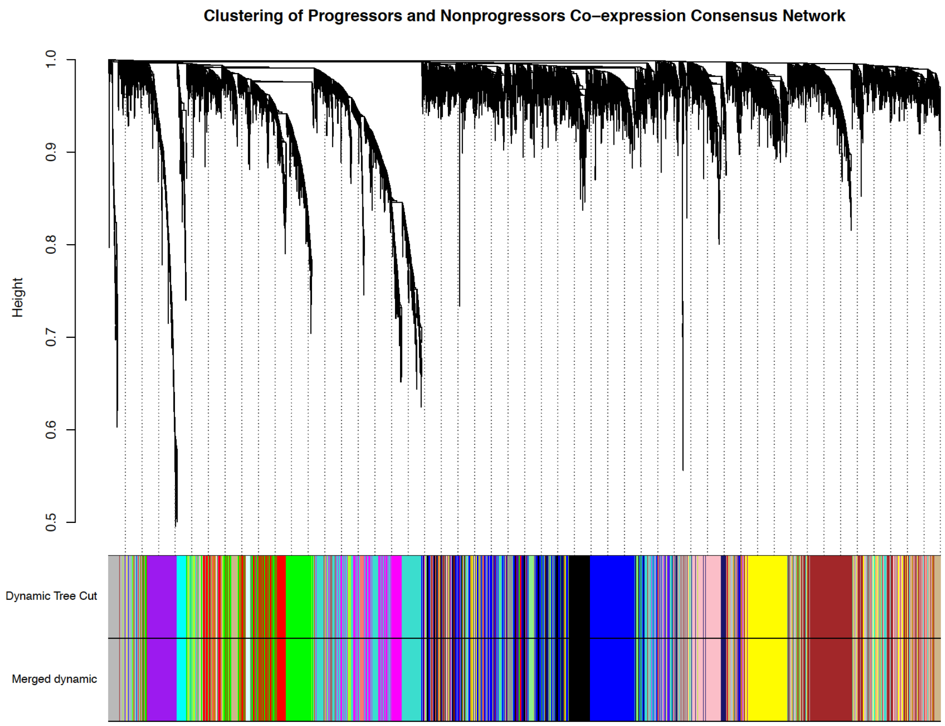

Figure S5: *Progressor and NonProgressor Consensus co-expression network (B) RNA_SeqV2 normalized counts and log2 (x+1) transformed gene expression* dynamic clustering and module definition denoted by first color-band. Second color band studies the need of merging similar modules based on similarity of original modules eigengenes (1st principal component). Here merging was clearly unnecessary.

Table S1: Summary of auto block-wise consensus module detection with one block assigned to the total quantity of genes. Here we identified 18 proper modules with sizes ranging from 71 to 1389 genes and 882 unassigned (grey module).

Module	Black	Blue	Brown	Cyan	Green	Green yellow	Grey60
Size	541	1357	1065	182	688	350	71
Module	Light cyan	Light green	Magenta	Midnight blue	Pink	Purple	Red
Size	120	71	447	120	533	354	634
Module	Salmon	Tan	Turquoise	Yellow			
Size	193	262	1389	765			

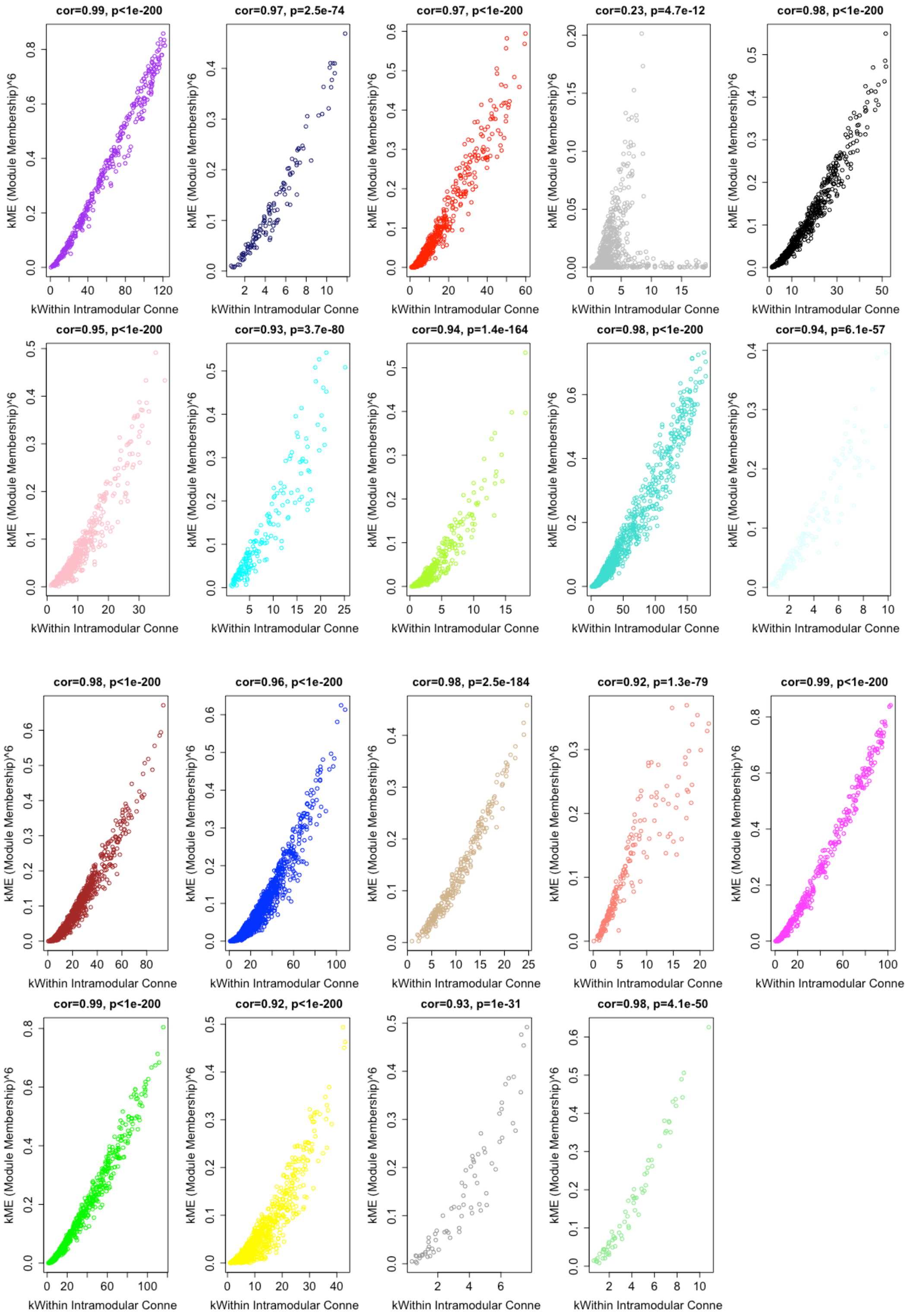

Figure S6: Consensus progressor module KME measures raised to the power of 6 (y-axis) vs. kWithin ($kIM_i = \sum_{i \neq j} a_{ij}$) intramodule connectivity (x-axis). Pearson correlation values between kME and KWithin of each module and corresponding student asymptotic p-value for a given correlation are demonstrated. Each dot represents a gene and is color-coded with its module accordingly. Here we conclude that all genes are properly assigned to modules based on kME and KWithin correlation and corresponding p-values (excluding the unassigned grey module)

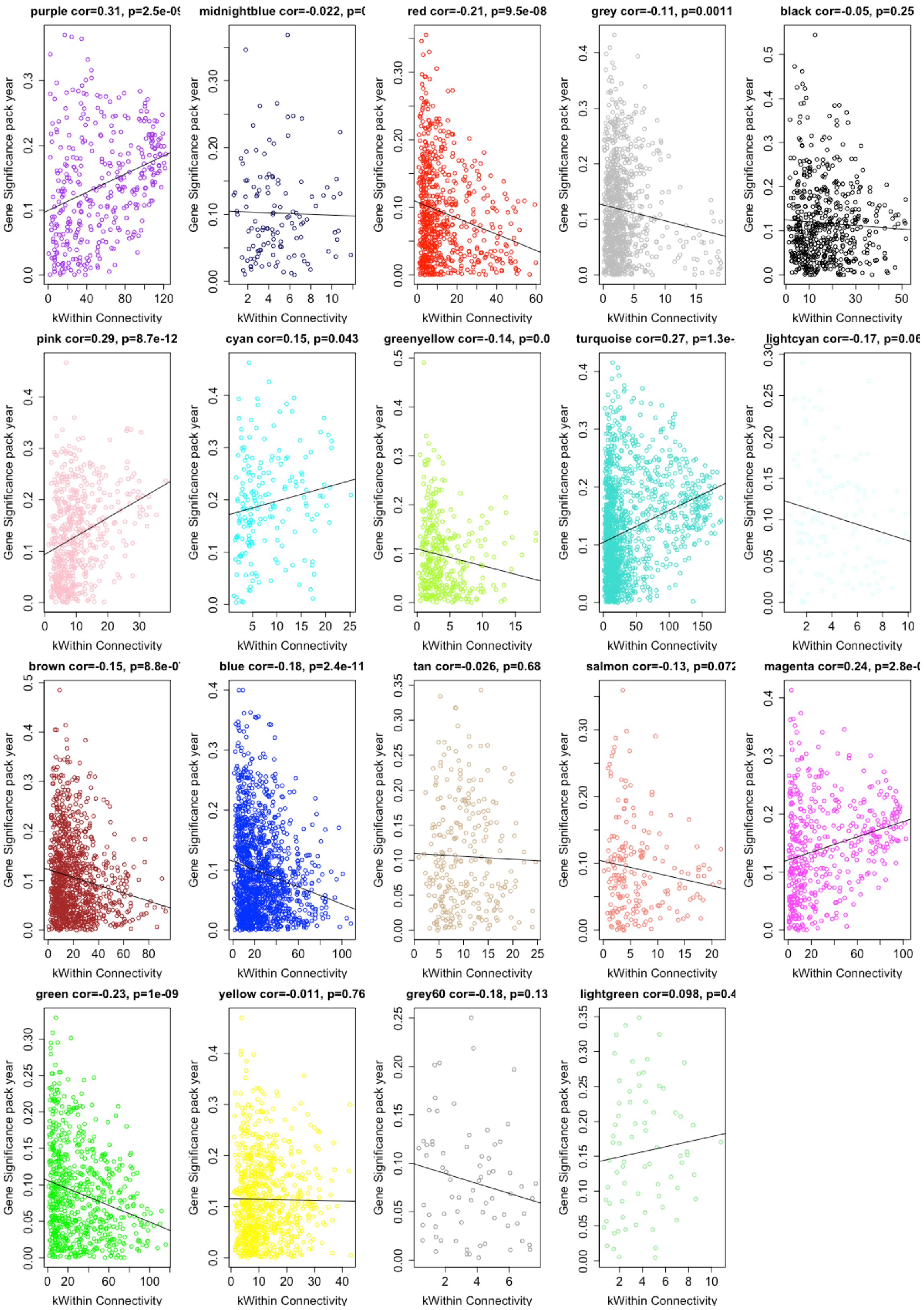

Figure S7: Scatterplot of each consensus module's gene significance$_{\text{pack-years}}$ (y-axis) vs. kME intramodule connectivity (x-axis) along with regression lines and corresponding Pearson correlations and p-values (student t-test). Each dot represents a gene profile and is color-coded by its corresponding module. Purple, pink, and cyan modules have the highest absolute value correlations and significant student t-test p-values between gene significance$_{\text{pack-years}}$ and kIM measures.

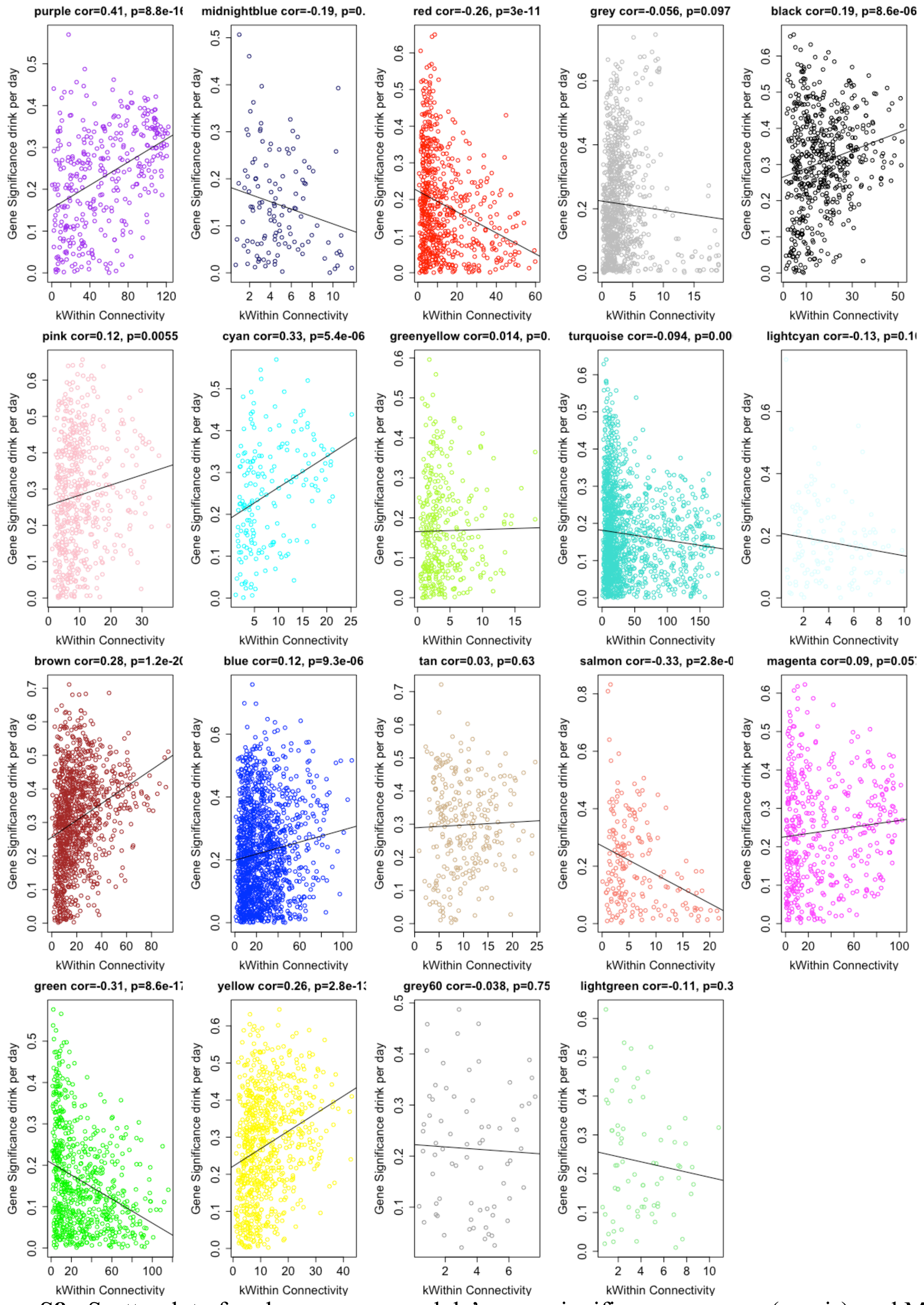

Figure S8: Scatterplot of each consensus module's gene significance$_{\text{drink per day}}$ (y-axis) vs. kME intramodule connectivity (x-axis) along with regression lines and corresponding Pearson correlations and p-values (student t-test). Each dot represents a gene profile and is color-coded by its corresponding module. Yellow, purple, cyan, brown, and black modules have the highest correlations and significant student t-test p-values between gene- significance$_{\text{drink per day}}$ and kME measures.

A

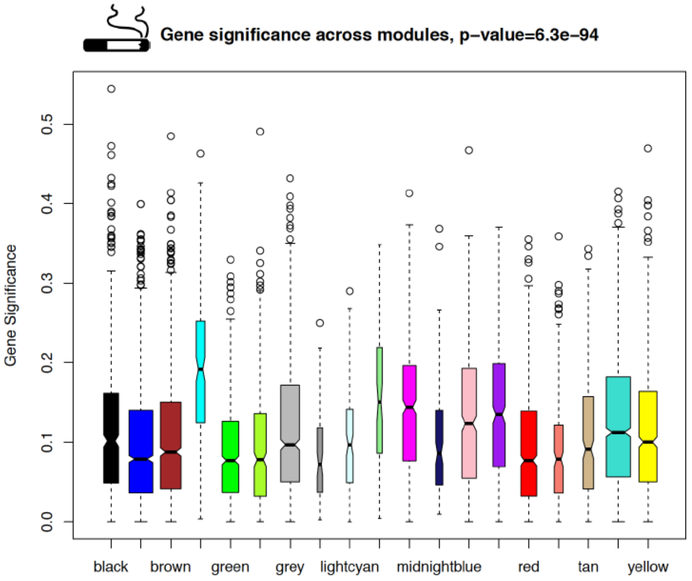

black	0.1177
blue	0.0966
brown	0.106
cyan	0.1924
green	0.0885
greenyellow	0.0958
grey	0.1169
grey60	0.0813
lightcyan	0.104
lightgreen	0.1562
magenta	0.1422
midnightblue	0.1021
pink	0.1302
purple	0.1377
red	0.0924
salmon	0.0915
tan	0.1056
turquoise	0.1249
yellow	0.1142

B

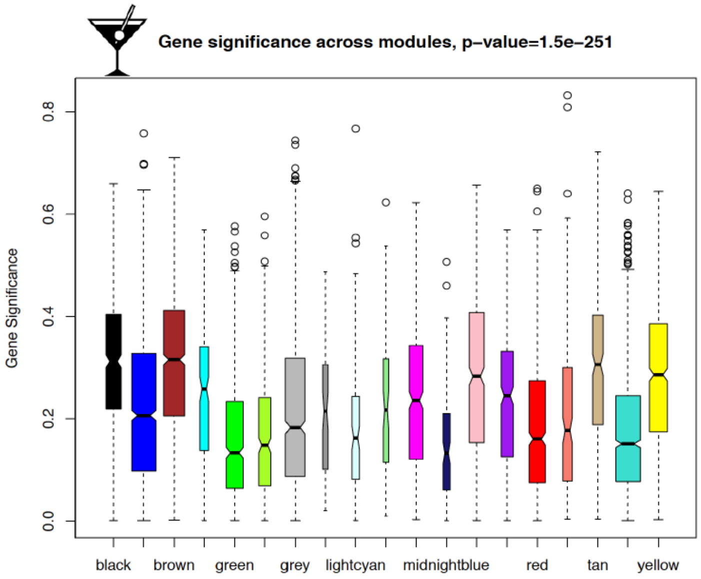

black	0.3065
blue	0.2235
brown	0.3081
cyan	0.2497
green	0.1604
greenyellow	0.1675
grey	0.2149
grey60	0.2144
lightcyan	0.1791
lightgreen	0.2308
magenta	0.2398
midnightblue	0.145
pink	0.2837
purple	0.2295
red	0.1859
salmon	0.2069
tan	0.2975
turquoise	0.1714
yellow	0.2789

Figure S9: A) Boxplot distribution of pack years' gene significance with each color-coded by the corresponding consensus module color (black, blue, brown, cyan, green, green yellow, grey, grey60, light cyan, light green, magenta, midnight blue, pink, purple, red, salmon, tan, turquoise, yellow) and table of module significance measures. The module significance here is defined as the average gene significance of the genes within a module. Gene significance of each gene is defined by the absolute Pearson correlations with tobacco pack years smoking clinical feature $GS_i = |cor(x_i, F_{pack-years})|$. The $GS = (GS_1, GS_2, \ldots, GS_n)$ measures are significantly different between modules (Kruskal Wallis test p-value: 6.3e-94). Only the cyan module showed the highest pack years' module significance (mean > 0.19). B) Boxplot distribution of drink per day gene significance and table of module significance mean measures. Gene significance of each gene is defined by the absolute Pearson correlations with drink per day alcohol consumption clinical feature $GS_i = |cor(x_i, F_{drink\_per\_day})|$. The $GS = (GS_1, GS_2, \ldots, GS_n)$ measures are significantly different between modules (Kruskal Wallis test p-value: 1.5e-251). Modules black, brown, cyan, pink, tan, yellow showed the highest module significance (mean > 0.25)

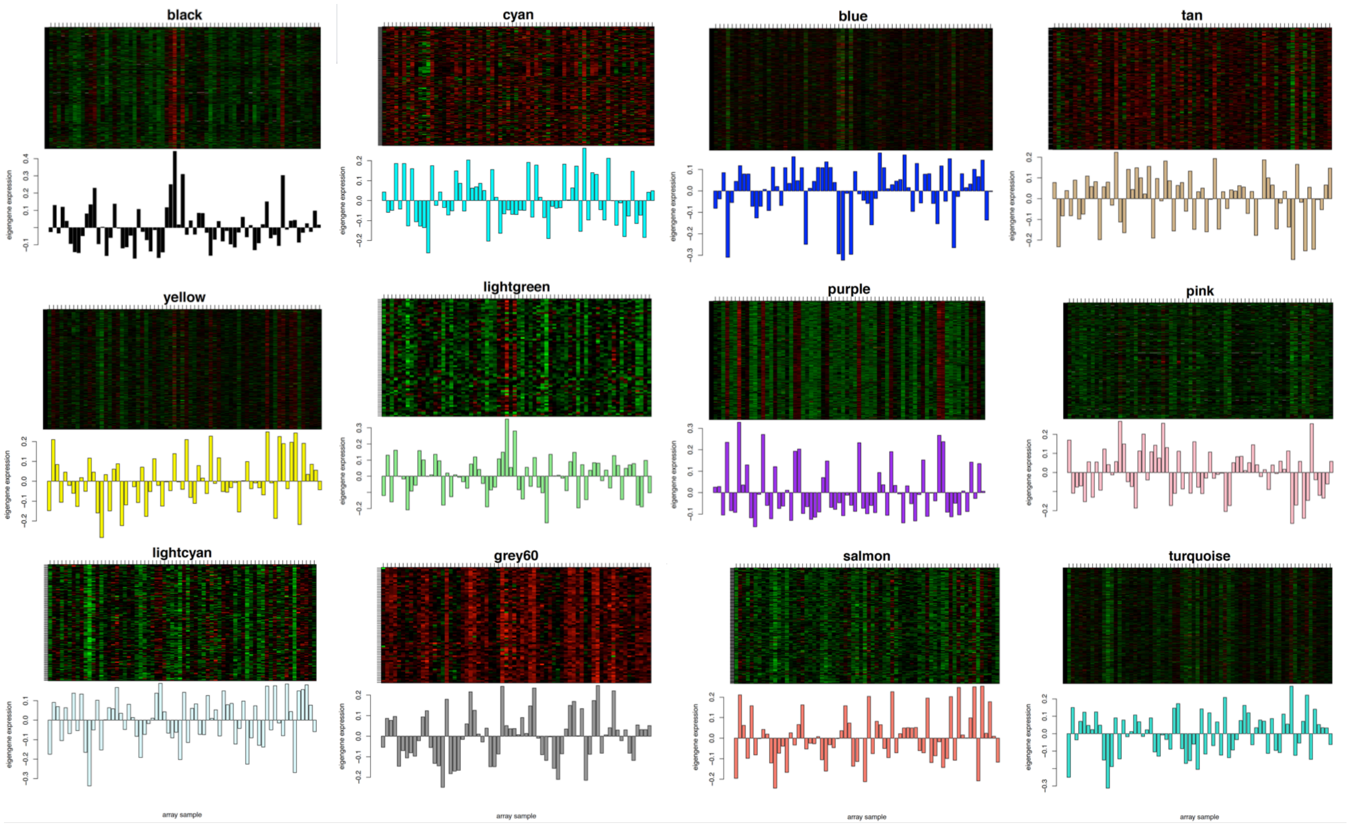

Figure S10: DE, DV, and DW consensus modules of progressor condition scaled ($scale = \frac{x-mean(x)}{sd(x)}$) gene X samples heatmaps and corresponding samples eigengene values barplot (color-coded by module membership). An essential unit of measure in WGCNA is eigengene (ME) that is closely defined as the 1st principal component. Principal components rely on scale dependency of variables and normalization is required ($scale = \frac{x-mean(x)}{sd(x)}$). Rows correspond to genes of consensus modules and columns correspond to progressor condition patients' samples. Heatmap colors red indicates high and green indicates low-scaled expression values. All consensus modules heatmaps of both conditions can be found in supplementary Data.
